# The development of powder profile refinement at the Reactor Centre Netherlands at Petten

**DOI:** 10.1107/S2053273317018435

**Published:** 2018-03-01

**Authors:** Bob van Laar, Henk Schenk

**Affiliations:** a Amsterdam, The Netherlands; b HIMS, FNWI, University of Amsterdam, PO Box 94157, Amsterdam, 1090 GD, The Netherlands

**Keywords:** powder profile refinement, profile refinement, Rietveld refinement

## Abstract

With thousands of references to ‘Rietveld refinement’ it is forgotten that the method did not suddenly appear in a flash of inspiration of a single person, but was the result of the work of three individuals working in the 1960s at the Reactor Centre Netherlands at Petten, Loopstra, van Laar and Rietveld. This paper outlines the origins of ‘profile refinement’, as it was called at Petten, and also looks at why it took so long for the scientific community to recognize its importance. With the recent passing of Hugo Rietveld, the death of Bert Loopstra in 1998 and before other pioneers also disappear, it is important to set down a first-hand account.

## Abstract 2

Around 1965 at the Reactor Centre Netherlands at Petten, Loopstra, van Laar and Rietveld developed ‘profile refinement’. Although Loopstra had the idea, van Laar worked it out mathematically and Rietveld wrote the computer program, the essential contributions of the first two are forgotten when using ‘Rietveld refinement’.

## 1. Introduction   

In the 1950s the Dutch government decided to set up a nuclear reactor centre in Petten, the Reactor Centre Netherlands, RCN. The construction started and some scientific co-workers were hired and sent abroad to learn how to work with the future equipment. On the list of future activities were neutron scattering and neutron diffraction, the latter being the focus of this article.

At the time the usual method of handling neutron powder patterns was to integrate the intensity of overlapping peaks and to use these compound intensities along with the intensities of single peaks in a classical structure-factor refinement with a program like *ORFLS* (Busing *et al.*, 1962 ▸). The function to be minimized was
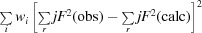
where 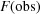 and 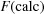 are the observed and calculated structure factors, *j* is the multiplicity of the reflection, the inner sum is over *r* overlapping reflections measured as one intensity, while the outer sum is over these *i* summed intensities, weighted by *w*, after correcting for Lorentz and polarization factors. The parameters to be refined are the overall isotropic temperature factor, the overall scale factor, any one of the individual isotropic temperature factors and all atomic coordinates.

Restrictions were due to the present state of computers. For example, in the early 1960s the storage of the Electrologica X1 was just 8192 words of 28 bits. Only by coding the program in machine language was it possible to refine up to 33 selected parameters simultaneously. Convergence was rather slow, with up to ten cycles required (see *e.g.* Loopstra & Boldrini, 1966 ▸).

## 2. The development of the neutron physics group of RCN   

The neutron physics group at RCN Petten originally consisted of one person only, Bert Loopstra, who got his training in crystallography with Carolina H. MacGillavry in Amsterdam and worked among others on scattering factors (Berghuis *et al.*, 1955 ▸). Loopstra was hired in 1955 by the group constructing RCN and sent to Kjeller, Norway, to practice neutron scattering work. In the same period Jaap Goedkoop, the scientific Director of RCN, was also in Kjeller (Goedkoop & Loopstra, 1959 ▸). Among others Loopstra used neutron powder diffraction in Kjeller to locate the hydrogen atoms in CaH_2_ (Bergsma & Loopstra, 1962 ▸).

Back in Petten Loopstra started to construct a neutron powder diffractometer for the reactor, to operate at 1.092 Å. In 1963 he solved the structure of orthorhombic U_3_O_8_ with this equipment (Loopstra, 1964 ▸).

**Figure 1:**
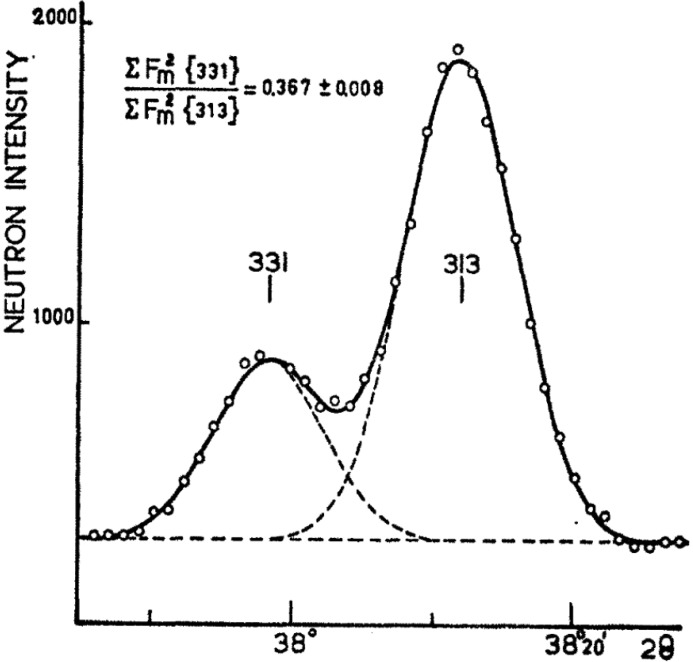
From van Laar (1965 ▸) illustrating his refinement of the profile of overlapping peaks to extract intensities.

In June 1960 Loopstra was joined at Petten by Bob van Laar, who also studied at the University of Amsterdam with Carolina MacGillavry, specializing in quantitative analysis. While he learned neutron scattering at Petten, he assisted with the setup of the neutron powder diffractometer, and started to specialize in the determination of magnetic structures by making himself acquainted with the calculation of the magnetic contribution to a neutron powder pattern. He refined in 1964 the magnetic structure of CoO (van Laar, 1965 ▸). In this compound too many pairs of peaks were overlapping and to resolve that problem their relative magnitude was found by performing a least-squares fit to their profile (see Fig. 1 ▸), with the assumption that the peaks were Gaussian as had been shown by Caglioti *et al.* (1958 ▸).

Similar attempts to extract the contributions of overlapping peaks had been made as soon as digital computers became available, *e.g.* at Argonne National Laboratory by Atoji & Williams (1961 ▸). It was later used in nuclear physics and spectroscopy, where it came to be known as ‘peak stripping’. Later, Pawley (1980 ▸) used a similar method that did not assume anything about the atomic structure apart from the lattice constants, which were refined along with the peak intensities.

Loopstra and van Laar shared an office. They engaged in many discussions, particularly about how to handle the problem of overlapping peaks. Van Laar had written down in detail the Caglioti formulae for how the peak shape varied with collimation, wavelength and scattering angle. Loopstra, the physicist, realized already in 1963 that the complete analytical powder profile, based on these formulae, should be used in the process of fitting the crystal structure to the observed profile. This turned out to be the basis later for the ‘profile refinement method’. At that time neither had any experience whatsoever with the programming of computers. So Loopstra carefully stored the idea of ‘whole profile use’ for the future.

Another idea of Loopstra’s was to tackle the overlap using a diffractometer with a much larger wavelength. This idea he realized by constructing a new powder diffractometer with a neutron wavelength of 2.6 Å (Loopstra, 1966 ▸), using a pyrolytic graphite filter to exclude shorter wavelengths (Bergsma & Van Dijk, 1967 ▸). This spread out the long *d*-spacing peaks, allowing more of them to be resolved, and is still a good solution for the magnetic structures in which the group were interested. However, for structure refinement too many peaks were still unresolved, and the shorter *d*-spacings, needed for high atomic resolution, could not even be seen.

When the group was allowed to employ a third member, it was obvious that computer expertise would be an advantage. So Hugo Rietveld was recruited at the end of 1964. He had been born in the Netherlands, but migrated to Western Australia with his family, where in 1957 he enrolled for a PhD at the University of Western Australia (UWA) at the same time as Brian O’Connor and Syd Hall. He obtained his doctorate in 1964 under the supervision of Ted Maslen. With C. J. B. Clews, Ted Maslen and Terry Sabine, he pioneered neutron crystallography with single crystals at Lucas Heights, and their first paper on diphenylbenzene was published in *Nature* (Clews *et al.*, 1961 ▸).

At Petten, Rietveld had to be converted from single-crystal crystallography to supporting the interests of the group in neutron powder diffraction and magnetic structures. In both fields he had neither knowledge nor experience as he had worked up to then on non-magnetic, single-crystal problems. However, as Rietveld’s expertise in computing filled the above-described gap in the knowledge of Loopstra and van Laar, it was a natural and welcome consequence for them to involve Rietveld in the profile refinement project.

As usual at the time, for his first powder experiment Rietveld used the sum of overlapping reflections in a least-squares refinement of structure factors as described in the introduction (§1 of this paper) (Rietveld, 1966 ▸).

## 3. Profile refinement – from idea to reality   

After Rietveld had settled into his new environment Loopstra explained his ideas for addressing the problem of overlapping peaks by using the complete observed profile of the powder pattern. van Laar described to him how the powder pattern depends on collimation and scattering angle, and contributed his formulae for calculating the magnetic contribution, unfamiliar to classical crystallographers.

With his computer experience, Rietveld then set about realizing the group’s dream for a better way to include the use of the observed profile instead of overlapping peaks in the refinement. In May 1966 the Electrologica X8 computer became fully available with its excellent ALGOL compiler, which made it possible to create a program that could use a large number of data points and refine all parameters. Using Loopstra and Boldrini’s 1966 data he published a short note (Rietveld, 1967 ▸, received by *Acta Crystallographica* on 28 July 1966) in which the observed intensity at each point in the profile 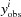 was written as the *w_i_*-weighted sum of the individual peak intensities (or squared structure factors 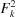): 
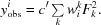
Curiously this paper does not emphasize the fact that structural parameters were not refined to fit the Bragg intensities but instead to fit the observed powder profile, which can be considered as a fundamental break with classical crystallography.

**Figure 2:**
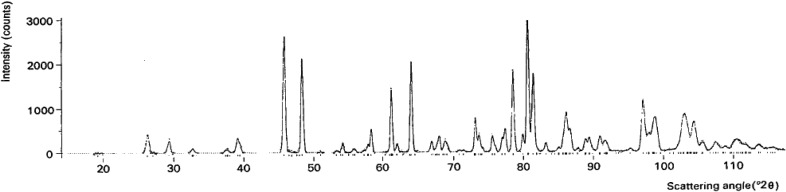
From Loopstra & Rietveld (1969 ▸) showing the ‘full profile’ refinement method, where the complete Sr_2_UO_5_ powder pattern is fitted directly by a structural model where the parameters are the atomic coordinates.

As soon as there was a working computer program Loopstra was going to use it, eager as he was to see how his idea improved refinement results. He had collected the best possible powder diagrams of seven alkaline-earth metal uranates in order ‘to determine the oxygen positions in Ca_2_UO_5_ and Sr_2_UO_5_ and to obtain better oxygen coordinates in the other compounds by using neutron diffraction data and the profile refinement technique’. He was delighted with the results as all objectives were reached. One of the results, Sr_2_UO_5_, is shown in Fig. 2 ▸. The article (Loopstra & Rietveld, 1969 ▸) was received by *Acta Crystallographica Section B* on 17 April 1968. In the section ‘Structure Refinement’, the new technique is explained in detail, although regrettably this has not been recognized by the scientific community. In our view this article marks the real start of profile refinement. If the community had awarded this (Loopstra & Rietveld, 1969 ▸) paper the recognition it deserved, the described profile refinement method would probably not be named after Rietveld alone. From the point of view of historical correctness this would have been much closer to the truth.

Rietveld wrote the technical description of the computer program in more detail, in which he also presented the mathematical background as given to him by van Laar. This paper arrived on 29 November 1968 at the IUCr office for the *Journal of Applied Crystallography* (Rietveld, 1969 ▸). With the more powerful computer all data for all peaks were included in the full profile refinement, with peak shape assumed to be Gaussian, their half width varying with the scattering angle according to the Caglioti *et al.* (1958 ▸) formula, their positions determined by the lattice dimensions, and their intensity determined by the atomic coordinates, which were refined directly to fit the full profile.

Although the effort at Petten to resolve overlapping peaks had taken several years, and included input from Loopstra for the idea of using the diffraction profile as the observation in the refinement procedure and from van Laar for the description of the magnetic contribution and the diffraction profile, Rietveld published his 1967 and 1969 computing papers as his own achievement, under his own name. He seemed to underestimate the scientific importance of the original idea of fitting the structure directly to the observed profile data and of the mathematical description of the process. The effort, the work and the idea of the two other participants were commemorated in one small sentence: ‘*The author wishes to thank Drs B. O. Loopstra and B. van Laar for their suggestions and helpful criticism*’.

Regrettably, the community marks this article as the start of the profile refinement method.

Rietveld (1969 ▸) elegantly described the difference between the conventional refinement of structure factors, the refinement of groups of overlapping reflections, and the profile refinement method, by comparing in each case the quantity *M* to be minimized. Using weights *W_i_*, structure factors *S_i_* and scale factor *c* for structure factors it is 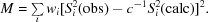
For the method using groups of *k* overlapping reflections:
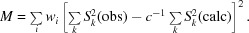
For the refinement of individual points *y_i_* of the profile it is
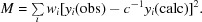
After publishing this important project alone, Rietveld found his position in the small Petten group increasingly difficult. In 1974 he successfully applied for the post of head of the RCN library, a function that had been vacant for some time, and consequently he left science. He remained with the library until his retirement in 1992.

In later years his point of view seemed to become more self-centred. An example can be found in Rietveld (2010 ▸) where he writes ‘*The arrival of computers with their immense computing power, gave me the idea that it should be possible to use the actually measured profile intensities instead of the derived integrated intensities*’.

## 4. How profile refinement developed   

Using Google Scholar to search for the citations to Rietveld’s 1967 and 1969 papers we found until 1973 only papers from the RCN and their relations in Kjeller, Munich and at PSI Switzerland. These concerned mainly magnetic structure refinements.

Although Rietveld had circulated his original ALGOL code widely, the method was seldom used. In those years Fortran became the winning compiler language and Rietveld rewrote his program also in Fortran II. When Alan Hewat visited RCN Petten in 1971, he brought back a copy of this version to Harwell, modified it to refine the anisotropic temperature vibrations found near structural transitions, and made it easier to use. And because Harwell had pioneered the concept of a ‘user facility’ with nearby Oxford University, the technique was then exposed to a wider scientific community and many new papers were published (Hewat, 1973*a* ▸,*b* ▸; Von Dreele & Cheetham, 1974 ▸).

In 1975 profile refinement really started to become known with 24 citations that year. At the neutron diffraction satellite meeting at Petten, associated with the 1975 IUCr Congress in Amsterdam, a debate was organized with the ‘father’ of European neutron crystallography, G. E. Bacon. Rietveld did not attend the meeting because he was treasurer of the Amsterdam congress. With Alan Hewat standing in, Bacon famously held up his notes proclaiming:

‘*Gentlemen, I have here the results of 60 years work in crystallography using single crystals*’ … (tearing up and dropping his notes) … ‘*and that is powder diffraction!*’

At the time it seemed an emphatic rejection, but in retrospect it was the greatest compliment a renowned crystallographer could make to the new method of profile refinement.

In 1976 the method crossed the Atlantic and was adopted by Von Dreele, Cox, Sleight, Worlton and Jorgensen. Worlton *et al.* (1976 ▸) used profile refinement for pulsed neutron data. In 1977 the first uses in X-ray diffraction were reported (Malmros & Thomas, 1977 ▸; Young *et al.*, 1977 ▸).

Citations of Rietveld (1969 ▸) and what Terry Sabine in 1978 christened in Cracow the ‘Rietveld method’ then grew exponentially, with new profile refinement programs by Taylor (1980 ▸), Wiles & Young (1981 ▸), and especially Larson & Von Dreele (1986 ▸) and Rodriguez *et al.* (1987 ▸). In 1977 it had already been applied to X-ray and neutron quantitative analysis (Werner *et al.*, 1979 ▸).

Manufacturers of X-ray powder diffractometers offer commercial quantitative analysis programs with their equipment, and users of these ‘black box’ methods often quote Rietveld (1969 ▸), even when their result has little to do with the original technique of refining atomic parameters to obtain a single structure. Quantitative analysis is more to do with refining the proportions of different phases contributing to the whole pattern, and other quantitative techniques naturally use similar computational methods. Indeed, many of the ∼12 000 who cite Rietveld (1969 ▸) have probably not read the paper, and do not need to.

## 5. Conclusions   

One of the most important ideas in powder diffraction is cited thousands of times as the description of a computer program. It would perhaps be more appropriate to cite instead the earlier Loopstra & Rietveld (1969 ▸) paper as the scientific basis and applications of the profile refinement method; it currently has only 0.9% the number of citations of the Rietveld (1969 ▸) paper. Rietveld left science before the importance of the work he had done with Loopstra and van Laar was recognized. Even later, profile refinement was confined to the relatively small community of neutron scatterers, before becoming a specialist tech­nique in X-ray powder diffraction. It was only really adopted by the wider crystallographic community 20 years later, after the original small group at Petten had all left science.

Of the Petten three, Loopstra succeeded MacGillavry in 1973 as professor in chemical crystallography at the University of Amsterdam and retired in 1987, published his last paper on superconductivity in 1989 (Westerveld *et al.*, 1989 ▸) and died in 1998 after a long illness. In 1985 van Laar left science, just after having published with Bill Yelon ‘*The Peak in Neutron Powder Diffraction*’ (van Laar & Yelon, 1984 ▸), a last contribution to the profile refinement method. He left science so completely that he did not even follow the field any more. 30 years later he was pleased that the work he contributed to had become so famous.

Thus when recognition finally did come, it was too late for these two scientists. Yet Rietveld, particularly after his retirement, was showered with personal honours – 1995, the Swedish Aminoff prize, 2003, the American Barrett award, 2004, the Dutch Officer in the Order of Oranje-Nassau, the 2010 International EPDIC Award, 2010, the German Hans-Kühl-Medal. Certainly the work is of the highest importance but perhaps it was inappropriate to honour only one person.

The crystallographic community, which had been slow to appreciate the significance of the work in the beginning, compensated decades later by lavish praise for ‘*one of the prominent crystallographers of the 20th century*’, ‘*for his distinguished achievement and insight…*’ and ‘*for his outstanding contribution to the field of chemistry*’. However, a simple survey of Rietveld’s full publication list shows that his pure crystallographic career was nothing more than moderate.

Rietveld’s own web page http://home.wxs.nl/~rietv025/ has an excellent record of the international recognition accorded for the invention of profile refinement, but it is difficult to find there any account of the scientific environment in Petten at the time, or acknowledgement of the contribution of others, in particular of the two other participants in the project.

## 6. Three historical notes   

(i) In his book in Dutch ‘De Republiek der Kerngeleerden’ (The Republic of Nuclear Scientists), about all science at the RCN, the Dutch scientific historian C. D. Andriesse (2000 ▸) concluded about the profile method:

‘*Hugo Rietveld, who was involved in the analysis of Diffraction, was the first to see the possibilities of a computer. Guided by the idea of Bert Loopstra and Bob van Laar that all the diffraction peaks would have the same (Gaussian) profile, he designed a numerical calculation code that was able to refine a measured diffraction pattern as well as possible into a number of peaks within that profile. This method has had a major impact, even outside the field of neutron diffraction. But because he published alone in 1969 it came to be known as the Rietveld method and the contribution of his counsellors was consigned to the dustbin of history*’ (translated from Dutch).

(ii) In 2001 one of us (HS) was invited to write about the profile method for a popular book ‘Chemie achter de Dijken’ (Chemistry behind the Dykes), to be published by the Royal Netherlands Chemical Society (KNCV) to celebrate the awarding in 1901 of the first Nobel Prize to J. H. van ’t Hoff. The brief was for two pages of text per finding or inventor, meant for a broad public. A well known science journalist was involved in the final editing to make it readable for the general public. The Rietveld method was selected as one of the citation champions of the century. So, summarized here, HS wrote in Dutch:

‘*Loopstra had the idea that it should be better to use the whole powder profile rather than estimated intensities to solve structures, van Laar worked it out mathematically and Rietveld programmed it. Nowadays it is known as the Rietveld method*’.

HS submitted the raw manuscript without figures and photographs. At the time he was President of the IUCr, very busy and travelling a lot. So when he saw the article again, it was already printed, there were four illustrations provided by Rietveld himself, and slight changes had been made to the text, which in essence had changed its meaning. In the sentence above Loopstra was replaced by Rietveld and it now started with ‘*Rietveld had the idea that…*’. Also a last sentence about Rietveld’s hobby, as a jazz pianist, had been added.

(iii) The present article is the third and last attempt to amend the description of the origin of the profile method. The authors hope and trust that by the present paper a more balanced picture of the origin of the profile refinement method will arise. It seems to us justified to replace the name ‘*Rietveld method*’ in the future by the working title of the past: ‘*profile method,*’ or to honour the inventor by using ‘*Loopstra method*’.

## References

[bb1] Andriesse, C. D. (2000). *De republiek der kerngeleerden (The Republic of Nuclear Scientists)*, p. 117. Betatext, Bergen (NH). ISBN 978-90-755-4105-2.

[bb2] Atoji, M. & Williams, D. E. (1961). *J. Chem. Phys.* **35**, 1960–1966.

[bb3] Berghuis, J., Haanappel, IJ. M., Potters, M., Loopstra, B. O., MacGillavry, C. H. & Veenendaal, A. L. (1955). *Acta Cryst.* **8**, 478–483.

[bb4] Bergsma, J. & Loopstra, B. O. (1962). *Acta Cryst.* **15**, 92–93.

[bb5] Bergsma, J. & Van Dijk, C. (1967). *Nucl. Instrum. Methods*, **51**, 121–124.

[bb6] Busing, W. R., Martin, K. O. & Levy, H. A. (1962). *ORFLS – A Fortran crystallographic least-squares program*. Report ORNL-TM-305. Oak Ridge National Laboratory, Tennessee, USA.

[bb7] Caglioti, G., Paoletti, A. & Ricci, F. P. (1958). *Nucl. Instrum.* **3**, 223–228.

[bb8] Clews, C. J. B., Maslen, E. N., Rietveld, H. M. & Sabine, T. M. (1961). *Nature*, **192**, 154–155.

[bb10] Goedkoop, J. A. & Loopstra, B. O. (1959). *Ned. Tijdschrift Natuurkd.* **25**, 1.

[bb11] Hewat, A. W. (1973*a*). *Nature*, **246**, 90–91. 10.1038/246090a0 4585852

[bb12] Hewat, A. W. (1973*b*). *J. Phys. C Solid State Phys.* **6**, 2559–2572.

[bb28] Laar, B. van (1965). *Phys. Rev.* **138**, A584–A587.

[bb13] Laar, B. van & Yelon, W. B. (1984). *J. Appl. Cryst.* **17**, 47–54.

[bb14] Larson, A. C. & Von Dreele, R. B. (1986). *GSAS*. LAUR 86-748. Los Alamos National Laboratory, New Mexico, USA.

[bb15] Loopstra, B. O. (1964). *Acta Cryst.* **17**, 651–654.

[bb16] Loopstra, B. O. (1966). *Nucl. Instrum. Methods*, **44**, 181–187.

[bb17] Loopstra, B. O. & Boldrini, P. (1966). *Acta Cryst.* **21**, 158–162.

[bb18] Loopstra, B. O. & Rietveld, H. M. (1969). *Acta Cryst.* B**25**, 787–791.

[bb19] Malmros, G. & Thomas, J. O. (1977). *J. Appl. Cryst.* **10**, 7–11.

[bb20] Pawley, G. S. (1980). *J. Appl. Cryst.* **13**, 630–633.

[bb21] Rietveld, H. M. (1966). *Acta Cryst.* **20**, 508–513.

[bb22] Rietveld, H. M. (1967). *Acta Cryst.* **22**, 151–152.

[bb23] Rietveld, H. M. (1969). *J. Appl. Cryst.* **2**, 65–71.

[bb24] Rietveld, H. M. (2010). *Z. Kristallogr.* **225**, 545–547.

[bb25] Rodriguez, J., Anne, M. & Pannetier, J. (1987). *STRAP. A system for time-resolved data analysis (powder diffraction patterns)*. ILL Report ILL87RO14T.

[bb27] Taylor, J. C. (1980). Australian Atomic Energy Commission Report AAEC/E488.

[bb9] Von Dreele, R. B. & Cheetham, A. K. (1974). *Proc. R. Soc. London Ser. A*, **338**, 311–326.

[bb29] Werner, P.-E., Salomé, S., Malmros, G. & Thomas, J. O. (1979). *J. Appl. Cryst.* **12**, 107–109.

[bb30] Westerveld, J. P. A., Cascio, D. M. R., Lo, Bakker, H., Loopstra, B. O. & Goubitz, K. (1989). *J. Phys. C*, **33**, 5689–5702.

[bb31] Wiles, D. B. & Young, R. A. (1981). *J. Appl. Cryst.* **14**, 149–151.

[bb32] Worlton, T. G., Jorgensen, J. D., Beyerlein, R. A. & Decker, D. L. (1976). *Nucl. Instrum. Methods*, **137**, 331–337.

[bb33] Young, R. A., Mackie, P. E. & von Dreele, R. B. (1977). *J. Appl. Cryst.* **10**, 262–269.

